# The Influence of Media Information Sources on Preventive Behaviors in China: After the Outbreak of COVID-19 Pandemic

**DOI:** 10.1155/2023/4941436

**Published:** 2023-04-08

**Authors:** Hongxiu Li, Li Pan, Weilu Chen

**Affiliations:** ^1^School of Tourism and Media, Chongqing Jiaotong University, Chongqing, China; ^2^Department of Media and Communication, University of Canterbury, Christchurch, New Zealand

## Abstract

**Background:**

As announced by the World Health Organization (WHO), since March 2020, the COVID-19 pandemic has become a global pandemic. In order to prevent the spread of COVID-19, Chinese government carried out very strict prevention and control policy.

**Objective:**

The study aimed to explore the effect of news reports on COVID-19 vaccine from traditional media and social media on COVID-19 preventive behaviors.

**Methods:**

Adults aged between 18 and 58 years old completed an online survey reporting how they gathered media information sources regarding the COVID-19 vaccine, as well as any details relating to risk perception, vaccine efficacy, and preventive behaviors in COVID-19 pandemic.

**Results:**

Our results showed that traditional and social media information sources both significantly and positively influenced people's COVID-19 preventive behaviors, with the former showing a stronger effect. COVID-19 contact risk perception and vaccine efficacy awareness of media audiences partly mediate this relationship. Audiences who reported more exposing news reports on COVID-19 vaccine from the media show stronger risk perception and vaccine efficacy awareness. This increases their COVID-19 preventive behaviors.

**Conclusions:**

This study found that media information sources have an important impact on people's COVID-19 preventive behaviors. People believe more in the news information of the mainstream media about the COVID-19 pandemic. Moreover, much of the news information of social media is also from the important mainstream media. Media organizations should shoulder greater social responsibility, embed the health-related benefits of COVID-19 vaccination into the values and cultural order of the whole society, find and shape a common space of meaning, and produce forms of internal coupling and value identification.

## 1. Introduction

The rapid spread and high mortality rate of the COVID-19 pandemic have caused panic and anxiety around the world. In this pandemic, news media coverage has an important role in preventing and controlling the spread of COVID-19 [1]. At the same time, people heavily rely on news media coverage for information when under complete lockdown [2]. Social media not only provides the public with important information regarding the COVID-19 pandemic but it can also influence government's policy-making when it comes to the progress and plans of pandemic prevention and control [3, 4].

Since the outbreak of the COVID-19 pandemic, China has been comprehensively reporting the pandemic through media platforms including CCTV, CGTN, and the *people*'*s daily*. Therefore, many Chinese residents perceive the degree of risk posed by the pandemic through news media coverage. China is the most populous country in the world, with a total of 1,443,497,378 people as of November 1, 2020. If the pandemic cannot be effectively controlled, the number of people infected with COVID-19 in China will rise rapidly. Vaccination, aimed at curbing the spread of the pandemic, is playing a vital role in China's control of the COVID-19 pandemic. By December 12, 2021, Chinese residents had received 2,612.218 million doses of vaccines [5]. If each Chinese resident receives two doses of the vaccines, approximately 1.3 billion people in China would have been vaccinated against COVID-19. Since November 2021, the Chinese government has been urging people to receive COVID-19 booster shots. Hence, Chinese residents who received two doses of vaccines half a year ago have received booster shots. As of December 13, 2021, the cumulative number of people infected by COVID-19 reached 129,135, and the cumulative number of deaths was 5,697, ranking the nation 108th among in the world. It is estimated that the incentive to be vaccinated is influenced by news media coverage in China. Additionally, according to the information we acquired, no scholars have explored the pandemic risk perception, vaccine efficacy awareness, and preventive behaviors from the perspective of news media coverage in China.

Therefore, it is particularly important to investigate people's opinions on pandemic risk perception, vaccine efficacy awareness, and preventive behaviors. To study the relationship between news media coverage and people's preventive behaviors, a nationwide survey on the risk perception, vaccine efficacy awareness, and preventive behaviors of the COVID-19 pandemic was conducted; our study population was adults in China. In this paper, we present more extensive research that highlights the correlation between news media coverage of the COVID-19 pandemic and sociodemographic preventive behaviors.

## 2. Methods

### 2.1. Research Design

Participants were recruited through Credamo, an online survey platform. As an international data platform, Credamo has registered users in all provinces, autonomous regions, and municipalities directly under the governance of the central government of China. We obtained informed consent from the participants and provided information stating that we would use the data for research purposes only.

### 2.2. Participants

Participants in this study include all urban residents aged 18 or above in all 31 provinces (and autonomous regions and municipalities directly under the governance of the central government) of China.

### 2.3. Questionnaire Design

In this study, a questionnaire that contains demographic characteristics and measures the media exposure of the Chinese news audience, pandemic risk perception, vaccine efficacy awareness, and preventive behaviors was designed based on the scales developed by Majid et al. [6]. The scales developed by local Chinese scholars for COVID-19 vaccine news media coverage, such as media exposure, risk perception, and preventive behaviors are taken from Ren et al. [7, 8].

This questionnaire was reviewed and approved by the Chinese research ethics review based on Credamo, an online survey platform.

### 2.4. Analysis Method

In this study, descriptive statistics were used to analyze the data, and SPSS 24.0 was used for correlation analysis between variables.

### 2.5. Measures

In this study, maturity scales were adopted and some English scales have been translated from English to Chinese. Each question in the scales was measured using a 5-point Likert scale, with “1” indicating “strongly disagree” and “5” indicating “strongly agree.”

#### 2.5.1. COVID-19 Contact Risk Perception

Risk perception was measured using the scales used by four questions on the 5-point Likert scale (“I think COVID-19 is a serious health issue,” “I think COVID-19 is bad for people's health,” “I think COVID-19 is a serious threat to my health,” and “I think COVID-19 is a serious disease”) which were used to measure the risk awareness of respondents.

#### 2.5.2. Vaccine Efficacy Awareness

Vaccine efficacy awareness was measured using the scales used by five items on the 5-point Likert scale (“I feel safe after being vaccinated,” “I can rely on vaccines to prevent serious diseases,” “I feel protected after being vaccinated,” “Vaccines have protected my family,” and “Vaccines have safeguarded public safety”). These items were presented in all regions, each presenting possible vaccine efficacy awareness regarding the coronavirus disease. Answer options ranged from 1 (do not agree at all) to 5 (fully agree).

#### 2.5.3. Preventive Behaviors

Preventive behaviors were measured using the scales [7] with the total four items (“Wear a mask when going out,” “Wash both hands frequently,” “Open windows for ventilation,”and “Prompt disinfection”). Answer options ranged from 1 (do not agree at all) to 5 (fully agree). These factor scores were saved and used in subsequent analyses.

#### 2.5.4. COVID-19 Traditional Media Information Sources

Three items were used to assess which channels were used by respondents to gather traditional media information sources [7] about the COVID-19: state-controlled news media (Xinhua News Agency, CCTV, People”s Daily, People”s Daily Online, etc.), local news media (local TV stations and local newspapers), and commercial news media (*Southern people weekly*, *Southern Metropolis daily*, *the paper*, *caixin weekly*, etc.). Three questions on the 5-point likert scale were used to measure the COVID-19 traditional media information sources, and answer options ranged from 1 (never) to 5 (mainly/always).

#### 2.5.5. COVID-19 Social Media Information Sources

Three items were used to assess which channels were used by respondents to gather social media information sources [7] about the COVID-19: WeChat official accounts, WeChat moments, and Weibo. For each mode of information sources with three questions, answer options ranged from 1 (never) to 5 (mainly/always). In the descriptive analyses, mean scores of these components were used for ease of interpretation.

### 2.6. Analytic Plan

First, descriptive statistics and bivariate correlations among study variables were presented. Afterwards, two multivariate regression models were tested, including as traditional media information sources relevant factors with COVID-19 contact risk perception, vaccine efficacy awareness and preventive behaviors, as social media information sources relevant factors with COVID-19 contact risk perception, vaccine efficacy awareness and preventive behaviors (derived from the correlational analysis). In order to further verify our conjecture, this research used regression analysis [9] test the mediating effect of COVID-19 contact risk perception and vaccine efficacy awareness on COVID-19 traditional/social media information sources and preventive behaviors.

## 3. Results

### 3.1. Descriptive Statistics

With the assistance of the online survey platform, 2,560 people were recruited and participated anonymously. Participants who completed the survey could receive a random reward from the platform, and the fee for each questionnaire was about RMB 4 (USD 0.57). We collected 1,429 survey forms. Through strict rejection procedures (e.g., rejection of forms with abnormal personal information, excessively short fill-in time, answers showing obvious regularity in choice of option), we finally obtained 995 valid questionnaires. The participants were urban residents, aged 18 to 58; (average age 28.97); from 31 provinces (and autonomous regions and municipalities directly under the governance of the central government) in mainland China. Among them, as shown in Table 1, 42.2% were male, 96% are nonmedical-related workers, 97.5% or more held an associate degree or above, 61.8% are married, 96.7% had been vaccinated against COVID-19, and 11.1% had cases of COVID-19 infection around them. It can be seen from the sociodemographic statistics that most of the participants were young people with an educational background above university level.

#### 3.1.1. Reliability and Validity Tests

In this study, maturity scales were adopted, and some English scales were translated from English to Chinese. Each question in the scales was measured using a 5-point Likert scale, with “1” indicating “strongly disagree” and “5” indicating “strongly agree.” Risk perception and vaccine efficacy awareness were measured using the scales used by Alabdulla et al. There were four questions for risk perception, with an internal consistency coefficient *α* of 0.61 and five questions for vaccine efficacy awareness, with an internal consistency coefficient *α* of 0.71. Preventive behaviors were measured using the scales used by Ren et al., with a total of five questions and an internal consistency coefficient of 0.68. The internal consistency coefficient of each variable in this study was above 0.6, which proves it to be reliable.

To confirm the discriminant validity of each variable, three variables (risk perception, vaccine efficacy awareness, and preventive behaviors) were subjected to the CFA test in this study. The three-factor model showed good data fitting (*χ*^2^ = 156.66, d*f* = 62, *χ*^2^/d*f* = 2.53, CFI = 0.96, TLI = 0.94, and SRMR = 0.04), indicating that this model represents the measured factor structure. In this way, the discriminant validity of risk perception, vaccine efficacy awareness, and preventive behaviors was validated.

#### 3.1.2. Risk Awareness

In this study, four questions on the 5-point likert scale were used to measure the risk awareness of respondents. The results found that the mean *M* of the respondents' risk perception was 4.41 (SD = 0.43), as shown in Table 2, showing a high-risk perception of respondents towards the COVID-19 pandemic.

#### 3.1.3. Vaccine Efficacy Awareness

In this study, five questions on the 5-point likert scale were used to measure the vaccine efficacy awareness of the respondents. The results found that the mean *M* of the respondents' risk perception was 4.29 (SD = 045), as shown in Table 3, showing that the respondents believed that COVID-19 vaccines were effective.

#### 3.1.4. Preventive Behaviors

In this study, four questions on the 5-point likert scale were used to measure the preventive behaviors of respondents. The results found that the mean *M* of the respondents' preventive behaviors was 4.24 (SD = 0.52), as shown in Table 4, showing that the respondents would take more active and enthusiastic preventive behaviors. It can be seen from the statistics that participants understood the routes of COVID-19 infection; for example, some people do not wear masks when going out, do not wash their hands, open windows, and do not disinfect frequently. Therefore, most participants knew that the main factors affecting the prevention behavior of COVID-19 were vaccines, masks, lifestyle, hand washing, social distance, disinfection, etc.

#### 3.1.5. Sources of News Media Coverage on COVID-19 Vaccines

The research findings show that both traditional and new media are the main fronts for COVID-19 vaccine news coverage. From Table 5, it is clear that the top four media platforms for participants to obtain information about COVID-19 vaccine are the Internet (93.9%), Weixin (81.5%), TV (79.8%), and Weibo (69.7%). It is clear that TV is the most important source of news media coverage in traditional media, while Weixin is the source of most news in new media. Some scholars believe that, in the face of the COVID-19 pandemic, China”s mainstream media, as the main body of information dissemination in major public health events, shouldered the responsibility of transmitting information, responding to social concerns, curbing the spread of rumors and guiding public opinions, and taking the initiative to publish information in a variety of ways; this approach has broken the long-term impact of new media and we-media on the ecology of public opinions and has established a good media image in the minds of audiences [10]. China”s mainstream media not only exert efforts in traditional media, but based on the concept of media convergence, still have a relatively high communication impact and influence in the fields of the Internet and mobile media [11].

#### 3.1.6. Audience”s Attention to COVID-19 Vaccine-Related Information

The mean for the question “How much attention do I pay to information related to COVID-19 vaccines?”, which was answered based on the degree of attention from very little to very much, is 4.35 (SD = 0.64), showing that the audience is very concerned about COVID-19 vaccine-related information. Specifically, 92.6% of the audience pay more attention to COVID-19 vaccine-related information and only 0.8% of the audience pay little attention to related information (see Table 6). Although most people will still pay more attention to information on COVID-19 vaccines, some scholars believe that compared with low-risk areas, familiarity and control of the public's risk perception in medium- and high-risk areas both have declined [11]. There are differences in the influence of the public's risk perceptions on their coping behaviors in risk areas. Therefore, there is still a portion of people who pays less attention to information on COVID-19 vaccines.

To further discuss the relationship between the audience's attention to information related to COVID-19 vaccines and their COVID-19 risk perception, vaccine efficacy awareness, and preventive behaviors, a Pearson's correlation analysis was conducted, and the results are shown in Table 7. It can be seen that the audience's attention to COVID-19 vaccine-related information is significantly correlated with their COVID-19 risk perception (*r* (993) = 0.20, *p* < 0.01), vaccine efficacy awareness (*r* (993) = 0.29, *p* < 0.01), and preventive behaviors (*r* (993) = 0.49, *p* < 0.01). The higher the audience's attention to COVID-19 vaccine-related information, the higher their COVID-19 risk perception and vaccine efficacy awareness, and the more intense their preventive behaviors. In addition, the COVID-19 risk perception, vaccine efficacy awareness, and preventive behaviors of the audience are positively correlated with each other.

#### 3.1.7. Frequency of Audience”s Exposure to COVID-19 Vaccine Information through News Media Platforms

According to the classification made by Ren Wei et al., in this study, media platforms are divided into six categories: state-controlled news media (Xinhua News Agency, CCTV, People”s Daily, People”s Daily Online, etc.), local news media (local TV stations and local newspapers), commercial news media (Southern People Weekly, Southern Metropolis Daily, The Paper, Caixin Weekly, etc.), Weixin official accounts, Weixin Moments, and Weibo. As shown in Table 8, 67.2% of the audience were most frequently exposed to state-controlled news media (*M* = 3.35, SD = 1.04), while 63.6% of the audience were most frequently exposed to Weibo and least frequently exposed to commercial news media (*M* = 2.18, SD = 0.94).

When it comes to the audience's exposure to each media type, it is apparent that the audience were most frequently exposed to state-controlled news media, with 475 (47.8%) audiences exposed to such media almost every day, followed by Weibo (471, 47.1%), Weixin Moments (417, 41.9%), Weixin official accounts (375, 37.6%), and local news media (262, 26.3%). The audience were least exposed to commercial news media, with only 100 (10%) audiences who are exposed to such media almost every day, as shown in Table 9. The result once again verifies that in the dissemination of COVID-19 information, China's mainstream media play a dominant role.

To further explore the frequency of the audience's exposure to COVID-19 vaccine information through news media platforms and their COVID-19 risk perception, vaccine efficacy awareness, and preventive behaviors, a Pearson's correlation analysis was conducted, and the results are shown in Table 8. It can be seen that the frequency of the audience's exposure to COVID-19 vaccine information is significantly and positively correlated with their COVID-19 risk perception, vaccine efficacy awareness, and preventive behaviors. As shown in Table 10, state-controlled news media (*r* (993) = 0.363, *p* < 0.01) and local news media (*r* (993) = 0.388, *p* < 0.01) are most correlated with preventive behaviors, and Weibo is least correlated with preventive behaviors (*r* (993) = 0.245, *p* < 0.01).

#### 3.1.8. Bivariate Correlations

Ren Wei divides media information sources into traditional media information sources and social media information sources [7]. Traditional media information sources include state-controlled news media, local news media and commercial news media, while social media information sources include WeChat official accounts, WeChat moments and Weibo. Our research adopts Ren Wei's classification method of media information sources to analyze the relationship between traditional media information sources and social media information sources and COVID-19 preventive behaviors.

Means, SDs, and correlation values among variables of interest are reported in Table 11. Due to the large sample size, correlation values above 0.06 (i.e., trivial in effect size) were significant at *p* < 0.05 [13]; Results showed that traditional/social media Information sources is significantly and positively correlated with COVID-19 risk perception, vaccine efficacy awareness, and preventive behaviors. Traditional media information sources (*r* (993) = 0.44, *p* < 0.001) and local news media (*r* (993) = 0.37, *p* < 0.001) are highly correlated with preventive behaviors.

Mean scores indicated that overall the traditional media information sources (*M* = 2.73) are less then social media information sources (*M* = 3.08). The COVID-19 contact risk perception (*M* = 4.41) showing a high-risk perception of respondents towards the COVID-19 pandemic, Respondents believe that COVID-19 vaccines have good effect (*M* = 4.29), and they would take more active and enthusiastic preventive behaviors (*M* = 4.24).

### 3.2. Multivariate Regression Models

**Figure 1:**
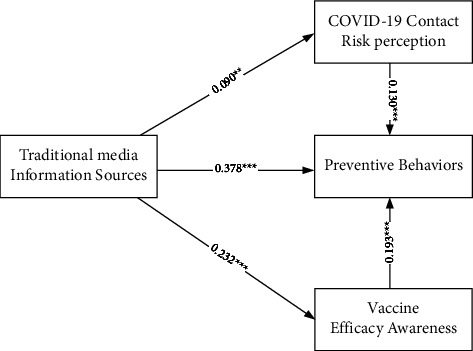
The multivariate regression models of traditional media information sources on preventive behaviors. ^*∗∗*^*p* < 0.01 and ^*∗∗∗*^*p* < 0.001.

**Figure 2:**
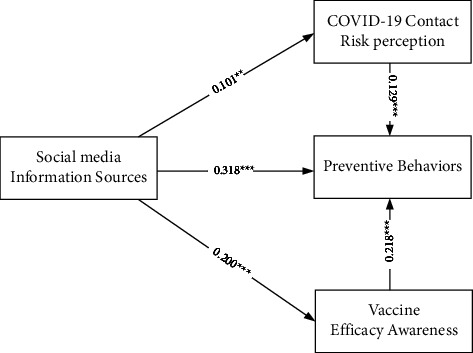
The multivariate regression models of social media information sources on preventive behaviors ^*∗∗*^*p* < 0.01 and ^*∗∗∗*^*p* < 0.001.

Standardized estimates of the two multivariate regression models are reported in Figures 1 and 2. The mediating effect test results are reported in Tables 12 and 13. In Figure 1, traditional media information sources are independent variables, preventive behaviors are dependent variables, and COVID-19 contact risk perception and vaccine efficacy awareness are intermediary variables. In Figure 2, social media information sources are independent variables, preventive behaviors are dependent variables, and COVID-19 contact risk perception and vaccine efficacy awareness are intermediary variables. The results showed that the mediation effect is established, COVID-19 contact risk perception and vaccine efficacy awareness partly mediates the relationship between traditional/social media information sources and preventive behaviors.

Figure 1 (see also Table 12) shows that traditional media information sources is significantly and positively influenced on COVID-19 risk perception (*β* = 0.090, *p* < 0.01), vaccine efficacy awareness (*β* = 0.232, *p* < 0.001), and preventive behaviors (*β* = 0.378, *p* < 0.001). COVID-19 risk perception (*β* = 0.130, *p* < 0.001) and vaccine efficacy awareness (*β* = 0.193 and *p* < 0.001) is significantly and positively influenced on preventive behaviors. It shows that COVID-19 contact risk perception and vaccine efficacy awareness partly mediates the relationship between traditional media information sources and preventive behaviors.

Figure 2 (see also Table 13) shows that social media information sources are also significantly and positively influenced on COVID-19 risk perception (*β* = 0.101 and *p* < 0.01), vaccine efficacy awareness (*β* = 0.200 and *p* < 0.001), and preventive behaviors (*β* = 0.318 and *p* < 0.001). COVID-19 risk perception (*β* = 0.129 and *p* < 0.001) and vaccine efficacy awareness (*β* = 0.218 and *p* < 0.001) is significantly and positively influenced on preventive behaviors. It shows that COVID-19 contact risk perception and vaccine efficacy awareness also partly mediates the relationship between social media information sources and preventive behaviors. Especially, the result that vaccine efficacy awareness is significantly and positively influenced on preventive behaviors is inconsistent with our past understanding.

Although results showed that COVID-19 contact risk perception and vaccine efficacy awareness both partly mediates the relationship between two types of media information sources and preventive behaviors. But the total effect of traditional media information sources on preventive behaviors (*β* = 0.434, *p* < 0.001) is stronger than social media information sources (*β* = 0.373, *p* < 0.001).

## 4. Discussion

The COVID-19 pandemic is a completely new and unexpected situation that is currently affecting many countries. COVID-19 is also considered a crisis or unexpected event given its tremendously harmful global impact [12]. Therefore, it is necessary to prepare a crisis management in short time period social changes. Otherwise, even a small-scale crisis will turn into a nationwide disaster, or even a worldwide issue. China was the most highly affected country at the time, with the pandemic spreading very fast. While the COVID-19 pandemic spread rapidly across the globe, media information sources are extremely important in preparing for mental and physical health issues. Media reporting not only pushes governmental framing and public policy during a public health crisis but it also provides leaders with data-empowered insights by using news and social media in relation to big data. On the contrary, misinformation narratives have serious consequences in terms of public health behavior and public safety [13], as well as decreased trust in governmental and health institutions [14]. The COVID-19 pandemic and media information sources were obvious, but the consequences of these on people's preventive behaviors were barely considered.

Our study is the first to examine the impact of COVID-19 pandemic media information sources on people's preventive behaviors. Based on news media coverage of COVID-19 vaccines in China, we explored COVID-19 risk perception, vaccine efficacy awareness, and preventive behaviors of a Chinese media audience. Follow-up discussions were held on the sources of news media coverage on COVID-19 vaccines, the audience's attention to information related to the COVID-19 vaccines, and the frequency of their access to COVID-19 vaccines information to further understand specific influencing factors. China media platforms can be divided into six categories: state-controlled news media (such as *Xinhua news agency*, *CCTV*, *people*'*'s daily*, *people*'*'s daily online*, etc.), local news media (local TV stations and local newspapers), commercial news media (such as *Southern people weekly*, *Southern Metropolis daily*, *the paper*, *caixin weekly*, etc.), WeChat official accounts, WeChat Moments, and Weibo. State-controlled news media are called mainstream media or traditional media, and WeChat official accounts, WeChat Moments, and Weibo are still called the new media or social media. The research findings show that both traditional and new media are the main fronts for COVID-19 vaccine news coverage. TV is the most important source of information in traditional media while WeChat is the most important source of information in new media. In the face of the COVID-19 pandemic, China”s traditional media shoulder the responsibility of transmitting by information, responding to social concerns, curbing the spread of rumors, and guiding public opinions. In other words, traditional media take the initiative to publish information in a variety of ways, which has broken the long-term impact of new media on the ecology of public opinions and established a good media image in the minds of audiences. China”s mainstream media not only exert efforts in traditional media but also have a relatively high communication impact and influence in the fields of the Internet and social media [11].

The effect we identified in our study can be explained in many ways. Although most people still pay more attention to information on COVID-19 vaccines [15], low-risk areas, such as familiarity and control of the public's risk perception, and high-risk areas have both declined. There are differences in the influence of the public's risk perceptions on their coping behaviors in risk areas. Traditional mainstream news or social media channels show the current social issues or latest trends linked with a tremendous shift in control toward individual citizens and groups in each country. Those media information sources impact the public risk perceptions, participatory actions, and audience engagement to preventive behaviors. This study shows the relationship between the audience's attention to information related to COVID-19 vaccines and their COVID-19 risk perception, vaccine efficacy awareness, and preventive behaviors. The higher the audience's attention to COVID-19 vaccine-related information, the higher their COVID-19 risk perception and vaccine efficacy awareness, and the more intense their preventive behaviors. In addition, the COVID-19 risk perception, vaccine efficacy awareness, and preventive behaviors of the audience are positively correlated with each other.

Our study found that mainstream media information sources are very important during the COVID-19 pandemic; however, compared with previous studies, our results demonstrate considerable differences. Previous studies have shown that social media are a significant information sources that influence attitudes toward COVID-19 vaccination [16]. In Qatar, social media has become an important tool for public communication and an important channel for the public to express their views anonymously. Of course, social media has also increased the public's negative attitude towards COVID-19 vaccine [6]. In Saudi Arabia, social media are the most important big data mining channels for information management regarding COVID-19 [16]. Chinese citizens also like to use social media to obtain information, but they are more confident in COVID-19 information reported by mainstream media. In the future, we will further study the relationship between preventive behavior and media information sources through cognitive theory [16].

## 5. Conclusion

When the world is fighting against the COVID-19 pandemic, we are also fighting against all kinds of false information about COVID-19 vaccines [12]. When all kinds of information flood around us, traditional media can play a leading role in public opinion during a global public health crisis. During COVID-19, people all over the world coincidentally believed in the information provided by traditional media rather than the information provided by social media [6], because social media is filled with many false information. In China, people believe more in the news information of the mainstream media about risk perception, vaccine effects, and preventive behaviors of COVID-19 pandemic. Moreover, much of the news information on social media is also from the important mainstream media, such as CCTV, Xinhua News Agency, People”s Daily, etc. Additionally, on the issue of COVID-19 vaccines, the frequent negative reports on the safety of COVID-19 vaccines abroad have also triggered public concern about the safety of vaccines [17]. Therefore, the government should be aware that social and cultural concepts constitute the “bottom public order” of healthy behavior [18]. In the process of health communication, the implementation of public health interventions cannot be separated from the social and cultural context. When the government publicizes and mobilizes the vaccination, it should embed the health concept of COVID-19 vaccination into the values and cultural order of the whole society, find and shape a common space of meaning, and produce forms of internal coupling and value identification.

## Figures and Tables

**Table 1:** Demographic statistics data of the respondents (*n* = 995).

Respondent types	Frequency (%)
*Gender*	
Male	420 (42.2)
Female	575 (57.8)
*Career*	
Medical-related workers	40 (4.0)
Company employees	647 (65)
Public servants	65 (6.5)
Teachers	63 (6.3)
Students	152 (15.3)
Others	28 (2.8)
*Educational background*	
Junior high school	3 (0.3)
Senior high school	22 (2.2)
Associate degree or above	848 (85.2)
Master”s degree or above	122 (12.3)
*Marital status*	
Unmarried	380 (38.2)
Married	615 (61.8)
*Vaccination status*	
Not vaccinated	33 (3.3)
Vaccinated	962 (96.7)
*Whether there is anyone infected with COVID-19 around*	
Yes	110 (11.1)
No	867 (87.1)
Unclear	18 (1.8)

**Table 2:** COVID-19 risk perception of the respondents.

Questions	Mean (standard deviation)
I think COVID-19 is a serious health issue	4.50 (0.60)
I think COVID-19 is bad for people's health	4.66 (0.51)
I think COVID-19 is a serious threat to my health	4.16 (0.74)
I think COVID-19 is a serious disease	4.33 (0.68)
Total average	4.41 (0.43)

**Table 3:** COVID-19 vaccine efficacy awareness of the respondents.

Questions	Mean (standard deviation)
I feel safe after being vaccinated	4.29 (0.71)
I can rely on vaccines to prevent serious diseases	3.91 (0.84)
I feel protected after being vaccinated	4.43 (0.63)
Vaccines have protected my family	4.31 (0.54)
Vaccines have safeguarded public safety (the safety of others)	4.49 (0.53)
Total average	4.29 (0.45)

**Table 4:** COVID-19 preventive behaviors of the respondents.

Questions	Mean (standard deviation)
Wear a mask when going out	4.62 (0.60)
Wash both hands frequently	4.19 (0.69)
Open windows for ventilation	4.31 (0.70)
Prompt disinfection	3.83 (0.88)
Grand mean	4.24 (0.52)

**Table 5:** Sources of news media coverage on COVID-19 vaccines.

Sources of coverage	Proportion of selected sources	Proportion of selected samples in total samples (%, *n* = 995)
TV	16.6	79.8
Newspapers	3.0	14.6
Radio	4.6	22.0
Internet	19.5	93.9
Weibo	14.5	69.7
Weixin	17.0	81.5
Short videos	13.1	62.9
Mobile app terminal	4.2	20.0
Other social media (QQ, Douban, Zhihu, etc.)	7.6	36.4
Total	100	480.8

**Table 6:** Frequency of audience's attention to COVID-19 vaccine-related information.

Degree of attention	Frequency (%)
Very little	1 (0.1)
Little	7 (0.7)
Does not care	65 (6.5)
Much	492 (49.4)
Very much	430 (43.2)

**Table 7:** Relationship between the audience's attention to COVID-19 vaccine-related information and their COVID-19 risk perception, vaccine efficacy awareness, and preventive behaviors.

	1	2	3
(1) Risk perception	—		
(2) Efficacy awareness	0.119^*∗∗*^	—	
(3) Preventive behaviors	0.186^*∗∗*^	0.296^*∗∗*^	—
(4) Attention	0.201^*∗∗*^	0.290^*∗∗*^	0.485^*∗∗*^

**Table 8:** Mean of the audience's exposure to COVID-19 vaccine information through news media platforms.

News media platform	Mean (standard deviation)
State-controlled media (Xinhua news agency, CCTV, p*eople*'*'s daily*, people”s daily online, etc.)	3.35 (1.04)
Local media (local TV stations and local newspapers)	2.66 (1.12)
Commercial news media (*Southern people weekly*, *Southern Metropolis daily*, *the paper*, *caixin weekly*, etc.)	2.18 (0.98)
Weixin official accounts	3.01 (1.12)
Weixin moments	3.05 (1.30)
Weibo	3.18 (1.30)

**Table 9:** Frequency of the audience's use to news media platforms.

News media platform	Hardly (%)	Once to twice a week (%)	Three to four times a week (%)	(Almost) every day	Several times a day (%)
State-controlled media (Xinhua news agency, CCTV, *people*'*'s daily*, people”s daily online, etc.)	28 (2.8)	202 (20.3)	290 (29.1)	338 (34)	137 (13.8)
Local news media (local TV stations and local newspapers)	155 (15.6)	338 (34)	240 (24.1)	212 (21.3)	50 (5.0)
Commercial news media (*Southern people weekly*, *Southern Metropolis daily*, *the paper*, *caixin weekly*, etc.)	267 (26.8)	403 (40.5)	225 (22.6)	82 (8.2)	18 (1.8)
Weixin subscription accounts	86 (8.6)	280 (28.1)	254 (25.5)	294 (29.5)	81 (8.1)
Weixin moments	146 (14.7)	230 (23.1)	202 (20.3)	269 (27.0)	148 (14.9)
Weibo	131 (13.2)	201 (20.2)	192 (19.3)	301 (30.3)	170 (17.1)

**Table 10:** Relationship between the frequency of the audience's exposure to COVID-19 vaccine information through news media platforms and their COVID-19 risk perception, vaccine efficacy awareness, and preventive behaviors.

	1	2	3
(1) Risk perception	—		
(2) Efficacy awareness	0.119^*∗∗*^	—	
(3) Preventive behaviors	0.186^*∗∗*^	0.296^*∗∗*^	—
(4) State-controlled news media	0.071^*∗∗*^	0.239^*∗∗*^	0.363^*∗∗*^
(5) Local news media	0.095^*∗∗*^	0.182^*∗∗*^	0.388^*∗∗*^
(6) Commercial news media	0.052	0.144^*∗∗*^	0.305^*∗∗*^
(7) Weixin official accounts	0.059	0.144^*∗∗*^	0.321^*∗∗*^
(8) Weixin moments	0.094^*∗∗*^	0.215^*∗∗*^	0.314^*∗∗*^
(9) Weibo	0.080^*∗*^	0.108^*∗∗*^	0.245^*∗∗*^

**Table 11:** Descriptive and bivariate correlations.

	Mean (SD)	1	2	3	4
(1) Traditional media information sources	3.01 (0.96)				
(2) Social media information sources	3.11 (1.09)	0.46^*∗∗∗*^			
(3) COVID-19 contact risk perception	4.41 (0.43)	0.09^*∗∗*^	0.10^*∗∗*^		
(4) Vaccine efficacy awareness	4.29 (0.45)	0.24^*∗∗∗*^	0.19^*∗∗∗*^	0.12^*∗∗∗*^	
(5) Preventive behaviors	4.24 (0.52)	0.42^*∗∗∗*^	0.33^*∗∗∗*^	0.19^*∗∗∗*^	0.30^*∗∗∗*^

^*∗∗*^ *p* < 0.01 and ^*∗∗∗*^*p* < 0.001.

**Table 12:** Mediating effect test (traditional media information sources).

	Preventive behaviors	Risk perception	Efficacy awareness	Preventive behaviors
Traditional media	0.434^*∗∗∗*^	0.090^*∗∗*^	0.232^*∗∗∗*^	0.378^*∗∗∗*^
Risk perception				0.130^*∗∗∗*^
Efficacy awareness				0.193^*∗∗∗*^
*R*-square	0.189	0.008	0.054	0.245
Δ*R*-square	0.188	0.007	0.053	0.243
*F*	230.776^*∗∗∗*^	8.177^*∗∗*^	56.679^*∗∗∗*^	107.292^*∗∗∗*^

^*∗∗*^ *p* < 0.01 and ^*∗∗∗*^*p* < 0.001.

**Table 13:** Mediating effect test (social media information sources).

	Preventive behaviors	Risk perception	Efficacy awareness	Preventive behaviors
Social media	0.373^*∗∗∗*^	0.101^*∗∗*^	0.200^*∗∗∗*^	0.318^*∗∗∗*^
Risk perception				0.129^*∗∗∗*^
Efficacy awareness				0.218^*∗∗∗*^
*R*-square	0.139	0.010	0.040	0.245
Δ*R*-square	0.139	0.009	0.039	0.243
*F*	160.907^*∗∗∗*^	10.158^*∗∗*^	41.231^*∗∗∗*^	86.048^*∗∗∗*^

^*∗∗*^ *p* < 0.01 and ^*∗∗∗*^*p* < 0.001.

## Data Availability

All the data and codes used in this study are publicly available at https://credamo.com/analysis.html?surveyld=6159#/survey-report.
